# Secure and Efficient Reactive Video Surveillance for Patient Monitoring

**DOI:** 10.3390/s16010032

**Published:** 2016-01-02

**Authors:** An Braeken, Pawani Porambage, Andrei Gurtov, Mika Ylianttila

**Affiliations:** 1 Department of Industrial Sciences and Technology (INDI), Vrije Universiteit Brussel, Brussels 1000, Belgium; an.braeken@vub.ac.be; 2 Centre for Wireless Communication (CWC), University of Oulu, Oulu 90014, Finland; mika.ylianttila@ee.oulu.fi; 3 Helsinki Institute of Information Technology (HIIT), Aalto University, Espoo 00076, Finland; gurtov@hiit.fi; 4 Department of “Secure Information Technologies” (SIT), ITMO University, Kronverkskiy prospekt 49, St. Petersburg 197101, Russia

**Keywords:** patient monitoring, visual sensor networks, security, privacy, authentication

## Abstract

Video surveillance is widely deployed for many kinds of monitoring applications in healthcare and assisted living systems. Security and privacy are two promising factors that align the quality and validity of video surveillance systems with the caliber of patient monitoring applications. In this paper, we propose a symmetric key-based security framework for the reactive video surveillance of patients based on the inputs coming from data measured by a wireless body area network attached to the human body. Only authenticated patients are able to activate the video cameras, whereas the patient and authorized people can consult the video data. User and location privacy are at each moment guaranteed for the patient. A tradeoff between security and quality of service is defined in order to ensure that the surveillance system gets activated even in emergency situations. In addition, the solution includes resistance against tampering with the device on the patient’s side.

## 1. Introduction

The rapid advancements of communication and sensing technologies have been deployed in many healthcare applications and ambient assisted living (AAL) systems. Due to the increasing elderly population and the desire of the general public for independent living, most of these systems are expected to be operated autonomously with minimum or no human intervention. Under such a circumstance, the monitoring applications of the caliber of video surveillance that are deployed in hospitals or retirement homes for patients and elderly people should always ensure their safety and privacy while maintaining operational efficiency and accuracy. Therefore, our proposed solution is applicable to those patients who are not immediately in a critical health situation, however being interested in following up on their health in a secure way, taking into account their privacy. From the moment a potential health issue occurs, the patient gets the opportunity to react. If the patient is not reacting (e.g., due to temporary inability), he or she is aware of the absence of that particular segment of security. However, the patient can still check and validate the procedures followed afterwards.

**Figure 1:**
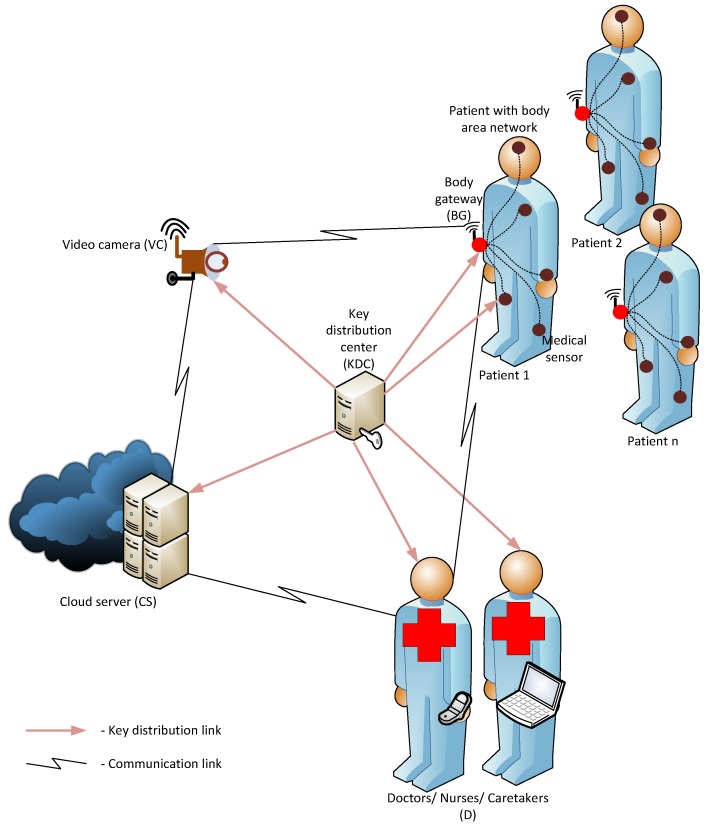
Network system architecture.

In this paper, we consider a conceptual design for the network architecture of a reactive video surveillance system in a medical environment, as illustrated in Figure 1. Accordingly, each patient *U* wears a wireless body area network (WBAN), which contains the medical sensors to acquire health records for different aspects and a body gateway (BG) node to aggregate and analyze the sensed data. The BG node also performs as a communicating entity from the WBAN to the outside network. Once a health-concerned or security threshold is notified by a particular medical sensor in the WBAN, the BG or the patient is responsible for invoking the video camera (VC) in closest proximity. The communication between the BG and VC should be activated by means of a private and authenticated request. The patient is in control of requests with a minor priority, while for urgent requests, no interaction of the patient is required. The privacy includes that no outsider can derive the identity of the person sending the request, whereas the authentication allows the submission of requests limited to people known to the system. Having a positive authentication validation, the VC captures the videos of the patient and sends them in the encrypted format to the cloud server (CS). The CS ensures the freshness of the data, stores the encrypted data and sends a notification about the newly-received encrypted video to the authorized persons (D) (e.g., doctors, nurses and caretakers). After that, the doctors with the corresponding credentials can decrypt the video and consult the patients accordingly.

For this particular network setting, we propose a secure video surveillance scheme that satisfies the following criteria:

- The scheme ensures the efficient involvement of all of the entities in the system with minimum power consumption and high performance.
- The patients’ user privacy and location privacy are guaranteed along with the data confidentiality.
- The VCs can be triggered manually by the patient or autonomously by the BG when the patient is unable to react. Since the second scenario does not guarantee the patient’s authentication, the VC and BG mutually authenticate and create user awareness and feedback.
- The solution is completely tamper proof at the patient level due to the construction of the key material. A leakage of security materials at the level of the VC is also detectable.
- The solution is flexible in terms of the patients’ password updates and the changes in doctor-patient relations.

The remainder of the paper is organized as follows: Section 2 provides a brief overview about the related previous work. Section 3 describes the threat model and the notations used. Section 4 explains the secure video surveillance scheme. Section 5 and Section 6 respectively discuss the security and performance of the proposed scheme. Finally, Section 7 summarizes the work and draws the conclusions.

## 2. Background and Related Work

In [1,2,3], the authors provide an extensive analysis of the wireless multimedia sensor networks and visual sensor networks along with their security and privacy threats and the protection mechanisms. According to [1], visual sensor network (VSN) security requirements can be classified into four key areas in terms of data, node, network and user-centric security. In data-centric security, it is expected to protect all forms of data (*i.e.*, raw and processed images or videos) made available by the visual sensors and ensure non-repudiation and confidentiality. Under data-centric security, privacy is identified as a sub-property of confidentiality, which indicates the protection of sensitive data against misuse by legitimate users.

In [4], Winkler and Rinner propose a user-specific and location-aware privacy awareness system integrated as an Android smartphone application. A distributed approach with the hybrid cryptosystem that ensures the confidentiality in a video surveillance system is presented in [5]. However, most of the security solutions proposed in the state of the art are for general purpose applications of video surveillance. Recently, numerous techniques have been proposed for the coordination and the control of multiple cameras, taking into account the potential loss of privacy [6,7,8]. Nevertheless, the key objectives of these mechanisms slightly deviate from the key scope of this paper. A complete security architecture for patient or elderly monitoring use cases is not particularly addressed in the previous literature. Although [9,10,11,12] specifically describe the VSNs in the context of assisted living and elderly care, they do not provide sufficient solutions for the security vulnerabilities, as discussed in [1].

A multitude of research on WBAN security and privacy already exists in the literature [13,14]. However, since the internal security of the WBAN is not the explicit focus of this paper, we here use a simplified security solution of a common shared group key among the sensors of the WBAN. Security solutions of eHealth systems, where the data of a WBAN is transmitted to the electronic records of the hospitals, has also been extensively studied. The most recent results propose adaptive security measurements [15]. However, by intuition, these solutions are in the first place too complicated to derive and secondly require complex implementations.

Our solution gives an answer to several identified challenges in [1]. First, we are able to create user awareness and feedback. We did not include user control of the data, since we believe that because of safety reasons, a doctor should be able to consult the data without waiting for patient approval. Second, we also define a solution that inherently satisfies tamper resistance from the patient side. The security mechanisms used in this paper are inspired from a protocol for biometric-based authentication with multi-server login using a smart card [16].

## 3. Threats and Notations

According to the network architecture, explained in Figure 1, several security threats can be encountered in the system as follows:

### 3.1. Security Threats

In [1], the following security threats in a video surveillance network can be distinguished.

- Illegitimate data access: An attacker tries to eavesdrop on the information exchanged in the network by, for instance overhearing the communication channel in order to use it for its own purposes.
- Illegitimate control access: An attacker tries to alter, insert, delete or change data sent in the network. In the worst case, an attacker is able to compromise one or several video cameras or body gateways in the network.
- Service degradation and denial of service: An attacker tries to reduce the availability of the network by, for instance jamming the wireless communication or by injection of a large amount of invalid requests. These attacks are often facilitated if the attacker has compromised one or several video cameras or body gateways in the network, as described in the previous attack.
- Malicious inside users: Here, special care needs to be taken, such that legitimate users do not leak any confidential information to outsiders. Minimal measurements to limit or trace this type of attack are the installation of a logging infrastructure.
- HW/SWattacks: An attacker tries to modify the configuration of the video camera or the body gateway in order to compromise the device.

It must be mentioned that complete protection against these types of attacks is inherently difficult. A minimal requirement is that detection mechanisms should be incorporated.

### 3.2. Notations

The most important notations and abbreviations used in the security scheme are described in Table 1.

**Table 1:** Notations used in the proposed security scheme.

Notation	Description
*x*	Master key of key distribution center (KDC)
*y*	Secret key shared among D’s and KDC
${ID}_{i},T_{i}$	Identity and nonce of patient *i*
${ID}_{D}$	Identity of the doctor D
$K_{G}$	Group key in WBAN
$K_{D}$	Secret key between D and KDC
*K*	Key to encrypt video by VC
*V*	Identifier for all VCs
*c*	Control status of message
$RPW_{i},A_{i},B_{i},C_{i},D_{i},E_{i}$	Security material related to $ID_{i}$
x∥y	Concatenation of *x* and *y*
H()	One-way hash function, e.g., Secure Hash Algorithm 3 (SHA3)
$E_{K}\left(\right)$	Symmetric authenticated key encryption using key *K*, e.g., AES-CCM

## 4. Overview of the Secure Video Surveillance Scheme

The proposed video surveillance scheme consists of six different phases. First, it starts with the initialization phase followed by a registration phase of the patients. For the sake of simplicity and the clarity of notations used, we restrict the explanations to one patient. The system architecture can accommodate multiple patients, where each patient will undergo the same procedure and receive similar forms of security materials with a unique identity. Then, the videos are captured, stored and processed respectively in the video recording, storage and decryption phases. Finally, the security materials are renewed in the update phase. Each phase is described in detail in the following sections.

### 4.1. Phase 1: Installation

The KDC contains one master key *x* and shares the following secret information with the different entities in the system, by means of physical contact or a secured channel.

- D: A common secret key *y* is shared with all of the doctors, together with distinct private shared secret keys $K_{D}=H(x∥y∥ID_{D})$. The identity of doctor D corresponds to $ID_{D}$.
- VC: The values H(x∥y), H(V∥H(x)) and H(V∥0) are shared with all VCs. The common identifier *V* is identical to every VC.
- Sensors in WBAN: A common group key $K_{G}$ is securely delivered to all of the medical sensors in the WBAN.
- BG: The group key $K_{G}$ and the identifier *V* are securely delivered to the BG.

### 4.2. Phase 2: Registration

This phase involves the registration of the patient and his or her WBAN into the system. First, the security materials should be derived for the BG based on the patient’s password. Then, the patient should establish the secure links with one or more doctors responsible for following up.

#### 4.2.1. Security Materials for BG

This phase requires physical contact between the patient and the key distribution center (KDC). The patient chooses a random value *b* and a password $PW_{i}$ and registers with the KDC using the information $ID_{i}$, $RPW_{i}\;=\;H\left(b⊕PW_{i}\right)$. The KDC computes the parameters $A_{i},B_{i},C_{i},D_{i}$ and $E_{i}$ as follows:

$$\begin{array}{ccc} A_{i} & = & H(y∥ID_{i}∥T_{i})\\ B_{i} & = & H\left(x∥y\right)⊕A_{i}\\ C_{i} & = & H\left(RPW_{i}∥ID_{i}\right)⊕H\left(A_{i}\right)\\ D_{i} & = & H\left(x\right)⊕H\left(ID_{i}\right)\\ E_{i} & = & RPW_{i}⊕ID_{i} \end{array}$$

Then, the BG stores the $B_{i},C_{i},D_{i},E_{i}$ parameters and the *b* value. Note that an attacker has no additional benefit when it is in possession of $B_{i},C_{i},D_{i},E_{i}$.

#### 4.2.2. Linking the Patient with the Doctor

The doctor(s) responsible for the care of patient $ID_{i}$ receives the parameter $ID_{i}$ together with a random value $T_{i}$ at the registration phase from the KDC. This information can be remotely sent as $E_{K_{D}}\left(ID_{i}∥T_{I}\right)$ using the shared key $K_{D}$ with the doctor. After that, the KDC eliminates all of the data derived for patient $ID_{i}$.

### 4.3. Phase 3: Video Request

The medical sensors in the WBAN sense data, encrypt them with $K_{G}$ and deliver them to the BG. Secure communication within the WBAN is not discussed in this paper, since it is out of the scope of the main scheme.

The BG analyzes the incoming data and invokes the alarms in two states according to predefined rules or threshold values. These states correspond to two different scenarios. In the first scenario (*i.e.*, medium alarm), the system requires the input of the identity and password of the patient. The second scenario (*i.e.*, urgent alarm) immediately starts sending a request. The medium alarm does not strictly require an input from the patient after two reminders. For the urgent alarms, the system can autonomously initiate the communication. Note that a patient might also have the possibility to request a video and/or an audio recording, even when there is no alarm. These situations all belong to Scenario 1. Both scenarios ensure the patient’s privacy, whereas the authentication of the request is only guaranteed in the first scenario. Finally, the last possibility is that there are three consecutive false inputs of the user ID and password, without the existence of an alarm. This situation might occur in the case of an attack and requires a change of password of the BG before any further action. The steps to perform here are discussed in Section 4.6.1. We now explain in detail the video request phase for both Scenarios 1 and 2 into detail.

#### 4.3.1. Scenario 1

Once the patient enters identity $ID_{i}$ and the password into the BG, it first computes $RPW_{i}$ using the inputs and the value *b* and then obtains $RPW_{i}⊕ID_{i}$. If this corresponds to the stored $E_{i}$, the patient is authenticated on the BG, and the process can be continued. Otherwise, a new trial should be undertaken by the patient using a random nonce $N_{i}$ generated by the BG as follows:

$$\begin{array}{ccc} H(x) & = & D_{i}⊕H\left(ID_{i}\right)\\ H(A_{i}) & = & C_{i}⊕H\left(RPW_{i}∥ID_{i}\right)\\ C_{1} & = & H\left(V∥H\left(x\right)\right)⊕H\left(ID_{i}∥N_{i}\right)\\ C_{2} & = & H\left(A_{i}\right)⊕N_{i}\\ V_{1} & = & H(N_{i}⊕B_{i})\\ CID_{i} & = & B_{i}⊕H\left(H\left(V∥H\left(x\right)\right)∥H\left(ID_{i}∥N_{i}\right)\right) \end{array}$$

The request to the VC consists of the message:

$$c∥CID_{i}∥V_{1}∥C_{1}∥C_{2}∥H(c∥CID_{i}∥V_{1}∥C_{1}∥C_{2}).$$

Note that control status *c* is a three-bit value, which is expected to be distinguished by the following possibilities.

- c=0: The user gave a correct input to the BG for a medium alarm.
- c=1: The user requests video and audio recording without a particular alarm situation.
- c=2: The user requests video recording without a particular alarm situation.
- c=3: The user requests audio recording without a particular alarm situation.
- c=4: The user did not react to the two alarms sent by the BG.
- c=5: The user gave the wrong input to the alarm of the BG.
- c=6: The alarm is urgent.
- c=7: The user gave the wrong input values more than three times.

Consequently, Scenario 1 corresponds to Control Statuses 1 to 3.

#### 4.3.2. Scenario 2

For Control Statuses 5 to 7, Scenario 2 is applied. As the patient does not enter the identity and the password, the authentication of the patient is not executed. Moreover, its security mainly depends on the stored value *V*, which means that the request can also be sent by compromised devices. Next, the same computations as before are performed, but now with H(x)=0, $ID_{i}=0$ and $H(A_{i})\;=\;0$. Consequently:

$$\begin{array}{ccc} H(x) & = & 0\\ H(A_{i}) & = & 0\\ C_{1} & = & H\left(V∥0\right)⊕H\left(0∥N_{i}\right)\\ C_{2} & = & N_{i}\\ V_{1} & = & H(N_{i}⊕B_{i})\\ CID_{i} & = & B_{i}⊕H\left(H\left(V∥0\right),H\left(0∥N_{i}\right)\right) \end{array}$$

The transmitted message also contains *c*, $CID_{i}$, $V_{1}$, $C_{1}$, $C_{2}$ and its corresponding hash.

### 4.4. Phase 4: Video Recording

When the message is received, the video cameras first check the integrity of the message by verifying the hash on the first part of the message. If this verification is successful, depending on the value of the control status, one of the two scenarios should be followed to check the authenticity of the message. We first describe the steps to be executed in the two scenarios as mentioned in Phase 3. Next, we explain which message should be sent from the VC to the CS.

#### 4.4.1. Scenario 1

The stored values H(V∥H(x)) and H(x∥y) are required to execute the following computations:

$$\begin{array}{ccc} H(ID_{i}∥N_{i}) & = & H\left(V∥H\left(x\right)\right)⊕C_{1}\\ B_{i} & = & CID_{i}⊕H\left(H\left(V∥H\left(x\right)\right)∥H\left(ID_{i}∥N_{i}\right)\right)\\ A_{i} & = & B_{i}⊕H\left(x∥y\right)\\ N_{i} & = & H\left(A_{i}\right)⊕C_{2}\\ V_{1}^{*} & = & H(N_{i}⊕B_{i}) \end{array}$$

If $V_{1}^{*}$ equals the transmitted $V_{1}$, the message is authenticated. A confirmation message, allowing mutual authentication, is sent to the BG. This message contains $\left(C_{3}∥V_{2}\right)$ and is calculated as:

$$\begin{array}{ccc} C_{3} & = & N_{j}⊕H\left(ID_{i}∥N_{i}\right)\\ V_{2} & = & N_{i}⊕H(H\left(V∥H\left(x\right)\right)∥B_{i}∥N_{j}) \end{array}$$

Having these values, the BG can derive $N_{j}=C_{3}⊕H\left(ID_{i}∥N_{i}\right)$. If $N_{i}⊕H(H\left(V∥H\left(x\right)\right)∥B_{i}∥N_{j})$ corresponds to the transmitted $V_{2}$, the BG knows that the request is successfully treated. If too many requests are unanswered, the BG sends an alarm to the patient and the system administrator.

#### 4.4.2. Scenario 2

For Scenario 2, similar operations are performed in a different order, taking into account some simplifications, such as $A_{i}=H\left(A_{i}\right)=0$ with the stored value H(V∥0).

$$\begin{array}{ccc} H(ID_{i}∥N_{i}) & = & H\left(V∥0\right)⊕C_{1}\\ B_{i} & = & CID_{i}⊕H\left(H\left(V∥0\right)∥H\left(ID_{i}∥N_{i}\right)\right)\\ A_{i} & = & 0\\ N_{i} & = & C_{2}\\ V_{1}^{*} & = & H(N_{i}⊕B_{i}) \end{array}$$

If $V_{1}^{*}$ equals the transmitted $V_{1}$, the validity of the request can be obtained. As long as the value *V* is shared only among valid and honest users, the user can be considered as a registered user (although he is not authenticated at the BG level). In order to detect the misbehavior of the system, a list containing $B_{i},C_{2}$ and *c* is stored at the VC, and the detection of a problem is sent to the KDC from the moment abnormal incidents occur.

Furthermore, a confirmation message, allowing mutual authentication, is sent to the BG. This message contains $\left(C_{3}∥V_{2}\right)$ and is calculated as:

$$\begin{array}{ccc} C_{3} & = & N_{j}⊕H\left(B_{i}∥N_{i}\right)\\ V_{2} & = & N_{i}⊕H(H\left(V∥0\right)∥B_{i}∥N_{j}) \end{array}$$

The BG can now derive $N_{j}=C_{3}⊕H\left(B_{i}∥N_{i}\right)$. If $N_{i}⊕H(H\left(V∥0\right)∥B_{i}∥N_{j})$ corresponds to the transmitted $V_{2}$, the BG knows that the request is successfully treated.

#### 4.4.3. Recording and Submission

The recording can be started when the request is accepted. The video *m* is encrypted using the key $K=H(C_{2}∥H\left(A_{i}\right))$. Note that in the second scenario, only $B_{i}$ is derived. In order to obtain the corresponding $A_{i}$, the BG computes $A_{i}=B_{i}⊕H\left(x∥y\right)$, using its stored secret H(x∥y).

The following message $C_{2}∥H\left(H\left(C_{2}∥H\left(A_{i}\right)\right)\right)∥E_{K}\left(m\right)$ is then sent to the cloud.

### 4.5. Phase 5: Storage and Decryption of Video

The server first checks for the uniqueness of $C_{2}$. All messages with the same $C_{2}$ should arrive in a limited time frame, as otherwise, it can come from a replay attack. Moreover, thanks to the usage of a fixed parameter, $C_{2}$, the videos related to the same event can be grouped.

The patients can check the video as they have temporarily stored the value $C_{2}$, and after entering the password and identity, also $H(A_{i})$ can be derived. In this way, the patient receives feedback on the recorded video. Note that even if the request was sent as an emergency alarm, where only $B_{i}$ is used, the video can only be encrypted after a proper authentication of the patient with the BG.

On the other hand, a notification of video reception is sent by the CS to all *D*’s of the system, together with the challenge $C_{2}∥H\left(H\left(C_{2}∥H\left(A_{i}\right)\right)\right)$. Any *D* who has the information $ID_{i},T_{i}$ can derive $A_{i}$ using its secret information *y*. Consequently, this authorized *D* can then compute $H(H\left(C_{2}∥H\left(A_{i}\right)\right))$. If the challenge matches, the *D* is able to decode the message with key $H(C_{2}∥H\left(A_{i}\right))$.

### 4.6. Phase 6: Updates of Secure Materials

#### 4.6.1. User Password Update

This can be easily executed by the user, without the involvement of the KDC or other entities in the system. New values of $C_{i}$ and $E_{i}$ are stored at the BG.

#### 4.6.2. Patient Update with Doctor

If the patient changes doctors, the patient should be removed from the doctor’s list. Moreover, new values of $A_{i},B_{i},C_{i},D_{i},E_{i}$ should be computed at the KDC since the registration random value $T_{i}$ is changed. The updated values of $B_{i},C_{i},D_{i},E_{i}$ for the BG can be remotely sent, as an attacker does not gain any knowledge with solely these values. The other doctors only need an update on the value of $T_{i}$, which can also be remotely sent in the encrypted format using the secret shared key $K_{D}$.

#### 4.6.3. Change in Patient and Doctor Relation

In fact, it is sufficient to update the list of patients, corresponding to the doctor. However, as the doctor also holds a long time secret *y*, it might be time to update all of the parameters. On the other hand, note that a doctor with knowledge of solely *y* is still unable to perform any disturbing activities in the system.

#### 4.6.4. Corrupted BG and Patient

In this case, the parameters H(x) and *V* are revealed. The only impact is that now, the video requests, corresponding to alarm Phase 2, can be sent without the guarantee that the user is a registered one. The privacy of the user in the request is still present, only the authentication cannot be verified any longer. If there is abuse of this situation, which is determined thanks to the registration of these events by the video camera or perhaps by the central server in the case of increased inactivity on certain requests, the system operator can still decide to update the parameter *V*.

#### 4.6.5. Corrupted Video Camera

If the system parameters H(x∥y), H(V∥0) and H(V∥H(x)) are leaked, any request can be monitored, and the data submitted can be manipulated. In addition, also requests with an urgent alarm can be sent. However, as the video camera has no direct link to the identity of the user, the privacy of the patient is still guaranteed.

Moreover, in most of the cases, the request is taken by several video servers in the neighborhood. Consequently, the origin of the corruption can be revealed when comparing the different inputs. Once detection is noticed, it is advocated to update the system parameters for all entities of the system.

## 5. Security Analysis

This section discusses in detail the security and privacy properties of the proposed video surveillance scheme and how it overcomes the threats mentioned in Section 3.

### 5.1. Illegitimate Data Access

The only entities in the system that are able to decrypt the message are the patient himself/herself and the corresponding doctors (or care takers). This follows from the fact that the data are encrypted by the video cameras by using a key only derivable by the involved parties.

### 5.2. Illegitimate Control Access

In both scenarios of medium and urgent alarm phases, only the registered users can send a valid video request. Even when the identifier *V* of the videos is revealed, the requests of a medium alarm phase can be solely derived by the registered users. Synchronized timers are not exploited due to the high level of resource consumption. As a result, there can be a possibility of replaying requests. Notification of replay with the same VC will be noticed on account of the storage of the temporary list of $B_{i},C_{2},c$ in the VC. However, since all of the VC’s share the same nonce *V*, the message might also be replayed to another VC.

Although, even if the messages that can create replay attacks are not detected by the VC, there is still the powerful server that will immediately detect a replay and continue with further actions. Due to the mutual authentication of the video request, the BG will receive an acceptance message from the video camera. If this is not followed by any request, the alarm can be notified by the BG, as well. Furthermore, without knowledge of the security material (e.g., H(x∥y) to derive $A_{i}$), no harm can come to the encrypted video.

### 5.3. Service Degradations and Denial of Service

Denial of service would be a potential danger for the video camera. However, the camera starts recording only when a valid request is sent. Due to the usage of a random nonce, the requests cannot be replayed without a notification by one of the entities, as mentioned before. Moreover, no heavy cryptographic operations are exploited in the system. On the other hand, suppose that many invalid messages are sent to the CS; the CS will notice that no actions are followed by either patients or doctors after forwarding the message and notifying of the problem.

### 5.4. Malicious Inside Users

Here, we can distinguish the BG in combination with a malicious user or a malicious doctor. None of these entities have enough knowledge to generate new accounts. Moreover a malicious doctor does not have the knowledge to generate a valid video request of another user. When a video is accidentally leaked, the log files need to be investigated in order to find the source.

### 5.5. HW/SW Attacks

The system finds its origin in providing security for smart cards without tamper-proof requirements. The same ideas can be applied here to the side of the BG. Even with knowledge of $B_{i},C_{i},D_{i},E_{i}$, an attacker has no further advantage, since the input of the identity and password of the user is required. As mentioned before, breaking into a video camera has a minor direct security impact on the users. Moreover, it can be quite easily detected. However, a compromised video camera requires a complete update of the system.

### 5.6. Privacy

Note that the video request contains the parameter $CID_{i}$. This is a dynamic reference, related to the hidden identity $B_{i}$ of the user. Consequently, no outsider can ever link the request to a certain patient, nor link those with other previous or pending requests. This also guarantees the location privacy of the patient. Moreover, as the video camera has no knowledge about the link between the real identity and the parameter $B_{i}$, even a malicious video camera cannot break the privacy of the patient.

## 6. Performance Analysis

The performance of the proposed security scheme is presented on behalf of the BG and the VC, as those are considered the most resource-constrained devices. Since all of the operations in the system are limited to XORs, hashes and symmetric encryption, the computational complexity of the proposed security solution is reasonably low.

The analysis is limited to the first scenario, which has slightly higher complexity. Denote the number of XORs by $N_{X}$, the number of hashes by $N_{H}$ and the number of authenticated encryptions by $N_{E}$. Table 2 summarizes the number of computations at the BG and the VC, for each valid request, leading to the recording and submission of the encrypted video.

**Table 2:** Performance of the body gateway (BG) and video camera (VC).

Phase	BG	VC
Video request	$6N_{X}+7N_{H}$	0
Video recording	$2N_{X}+3N_{H}$	$7N_{X}+8N_{H}+1N_{E}$

Moreover, for the required communication phases, the length of the messages in each phase is moderate enough. For instance, assuming the length of the parameters in the system to be 128 bits, Table 3 summarizes the length of the transmitted and received messages, corresponding to the video request and video recording phase at the BG and VC. Let ∥m∥ denote the length of the video.

**Table 3:** Communication length at the BG and VC.

	BG		VC	
	Phase 1	Phase 2	Phase 1	Phase 2
Transmission	80 bytes	–	–	64 bytes + ∥m∥
Reception	–	32 bytes	80 bytes	–

In order to provide further insight into the performance of the proposed surveillance scheme, we have quantified the energy consumptions on behalf of BG and VC entities, which are considered the most critical devices for power optimization. We implemented the corresponding cryptographic and control operations on a Libelium Waspmote platform [17] using Waspmote cryptographic libraries. Waspmote has an Atmega1281 microcontroller running at 8 MHz with 8 KB SRAM, 4 KB EEPROM and 128 KB flash memory. We measured the execution time (*t*) for individual operations on the BG and VC and thereby calculate the computation energy cost using formula U×I×t based on the execution time (*t*), the nominal voltage (*U*) and the current draw in active mode (*I*) on Waspmote sensors. The communication between the BG and VC was performed by the Bluetooth Low Energy (BLE) protocol. The captured video is uploaded by the VC over a 3G network via the Secure File Transfer Protocol (FTPS). Table 4 shows the energy consumption values for different operations at the BG and VC.

**Table 4:** Energy consumption at the BG and VC.

	Operation	Time (ms)	Energy (mJ)
BG	Process video request	25	1.575
	Transmit video process request to VC	25	4.095
	Receive confirmation from VC	21	1.793
	Authenticate VC	45	2.835
	Total at BG	116	10.298
VC	Receive request from BG	25	2.135
	Authenticate BG	47	2.961
	Transmit ACK to BG	21	3.440
	Total at VC	93	8.536
VC	Transmit 2-s video to cloud	19,438	38,876

We could observe that a single elliptic curve cryptographic (ECC) multiplication operation consumed approximately 13.365 mJ on the Waspmote platform (for secp160r1 elliptic curve (EC) domain parameters). However, the total energy consumptions (*i.e.*, including computation and communication) at each entity (*i.e.*, the BG and VC) are less than this value. This gives an implicit assurance that the proposed scheme is efficient, and it will outperform resource-less devices compared to the ECC-related schemes.

## 7. Conclusions and Future Work

A patient monitoring system by means of reactive video surveillance strictly requires dedicated security mechanisms in order to guarantee the privacy and confidentiality of the patient’s data. In this paper, we proposed a complete and highly efficient solution solely using symmetric key cryptographic primitives to establish this goal. The patient is aware of the video monitoring and receives the required feedback.

The proposed security architecture can be easily extended to obtain extra performance features or even stronger security properties. Below, we present some examples.

- The data measured in the WBAN can be securely sent to the central server by using the VC’s as gateways or edge routers. These edge routers could share the same security mechanisms as the VC’s, however, then with a distinct common identifier *E*.
- In many commercial systems, video surveillance is combined with voice communication. Consequently, if a smartphone takes the role of the BG, the doctor can call the patient if required.
- We did not add watermarking techniques to the side of the VC’s, since we assumed that one request is taken by different VC’s, and thus, the combined information reveals the location of recording. In order to guarantee the integrity and authentication of the data from a particular VC, a watermark could be added to the image encryptions.
- As mentioned before, the usage of time stamps could speed up the detection of replay attacks. However, special care needs to be given to secure the synchronization between the clocks of the different entities.
- An additional communication layer can be added while applying the multi-camera fusion techniques. This may increase the performance of the system drastically.

As future work, we intend to implement the same conceptual design in a real system to retrieve the opinion of patients and healthcare professionals.
